# P-2174. Development and Validation of the Y.E.L.L.O.W. Score: a Novel Prognostic Model for Yellow Fever-Related Acute Liver Failure

**DOI:** 10.1093/ofid/ofaf695.2337

**Published:** 2026-01-11

**Authors:** Carolina Vieira, Bráulio R G M Couto, Wanessa T Clemente

**Affiliations:** Hospital Evangélico, Belo Horizonte, Minas Gerais, Brazil; AMECI – Associação Mineira de Epidemiologia e Controle de Infecções, Belo Horizonte, Minas Gerais, Brazil; Universidade Federal de Minas Gerais - UFMG, Belo Horizonte, Minas Gerais, Brazil

## Abstract

**Background:**

A 2017-2018 Yellow Fever (YF) outbreak in southeastern Brazil saw several cases develop acute liver failure (ALF), emphasizing the importance of prognostic tools. Current scoring systems guide treatment and liver transplant decisions, but their use in YF-related ALF is uncertain. This study presents the Y.E.L.L.O.W. Score (Yellow Fever End-stage Liver and Organ Worsening Score) for estimating death probability in YF patients with ALF.

**Table 1:**
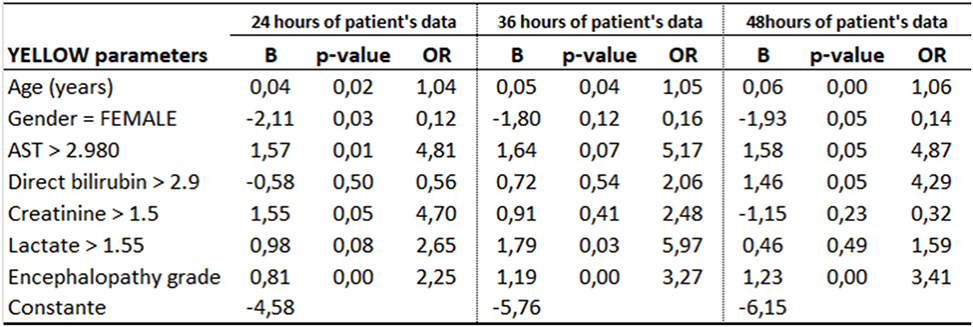
Logistic regression models for the Y.E.L.L.O.W. Score to predict death due to yellow fever. Logistic regression models for the Y.E.L.L.O.W. Score to predict death due to yellow fever.

**Figure 1:**
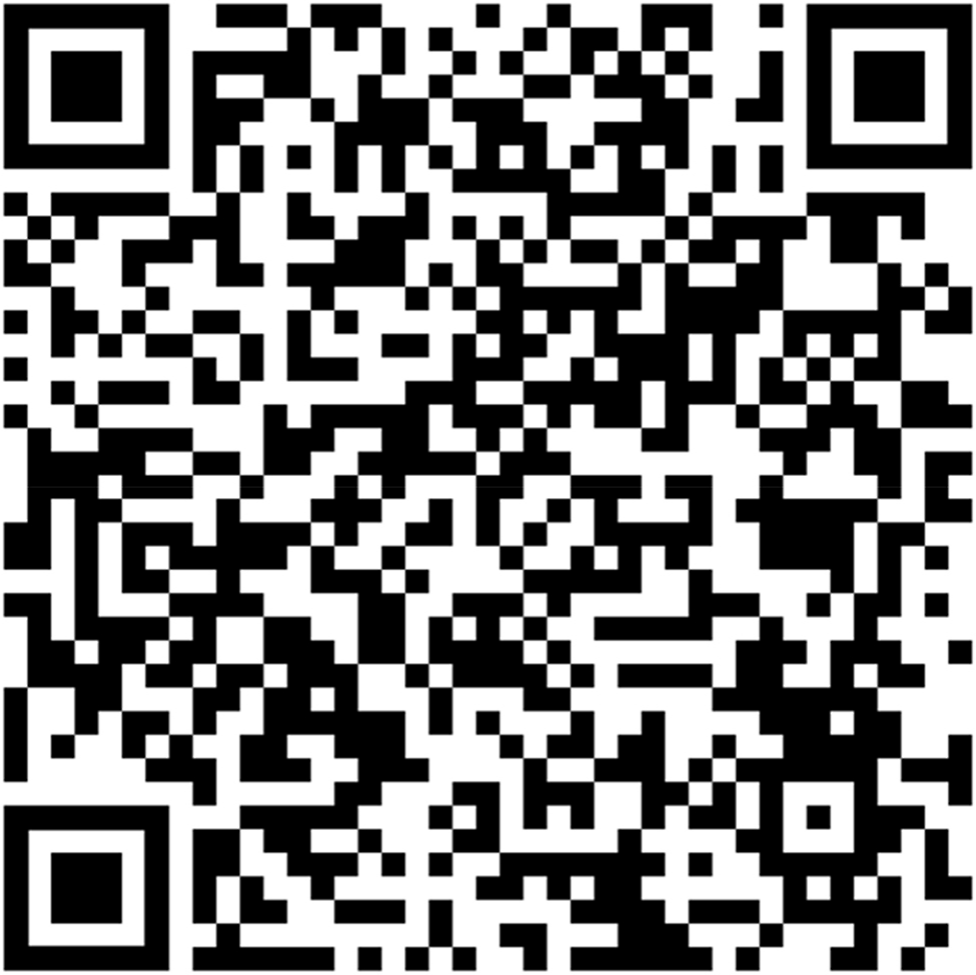
QR code linking to a spreadsheet that calculates mortality probability using one of the three Y.E.L.L.O.W. Score logistic regression equations. QR code linking to a spreadsheet that calculates mortality probability using one of the three Y.E.L.L.O.W. Score logistic regression equations.

**Methods:**

Data from 229 YF patients (confirmed by serology/PCR, age >18) admitted to a reference hospital in Minas Gerais, Brazil (Jan 2017-Mar 2018) were used to develop the Y.E.L.L.O.W. Score. Demographic data, in-hospital mortality, and lab results at 24, 36, and 48 hours were analyzed to estimate death risk. Logistic regression models assessed the Y.E.L.L.O.W. Score's predictive performance.

**Figure 2:**
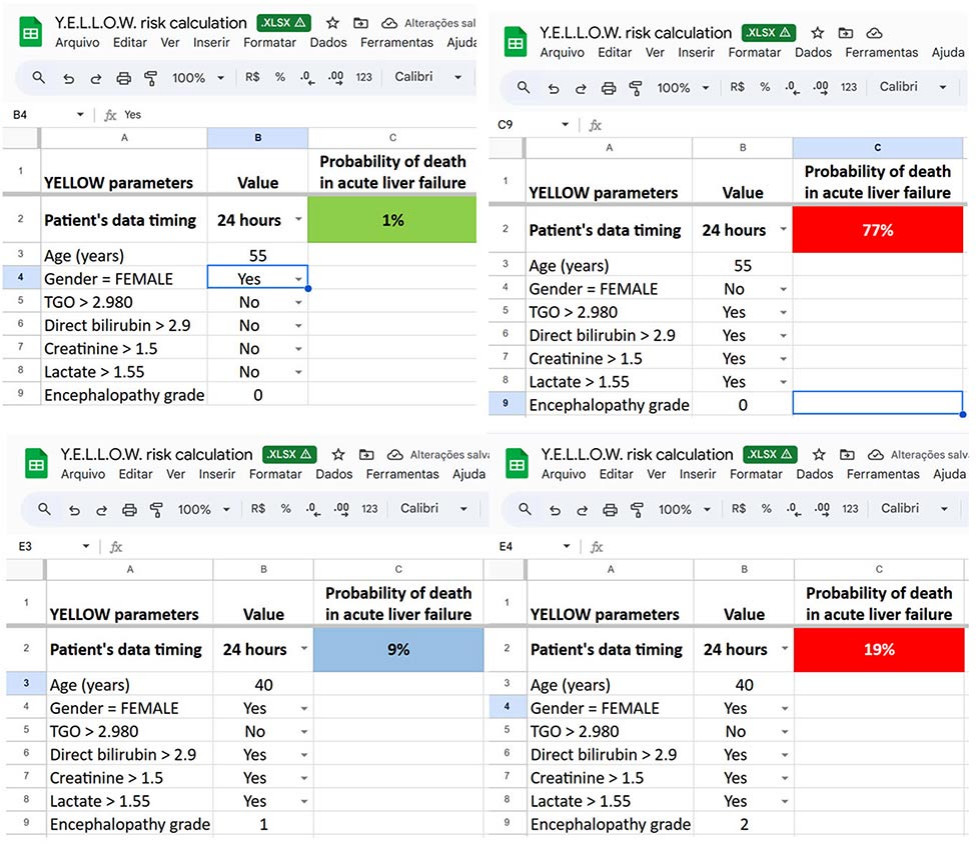
Examples demonstrating the use of the spreadsheet to calculate mortality probability with the Y.E.L.L.O.W. Score logistic regression equations. Examples demonstrating the use of the spreadsheet to calculate mortality probability with the Y.E.L.L.O.W. Score logistic regression equations.

**Figure 3:**
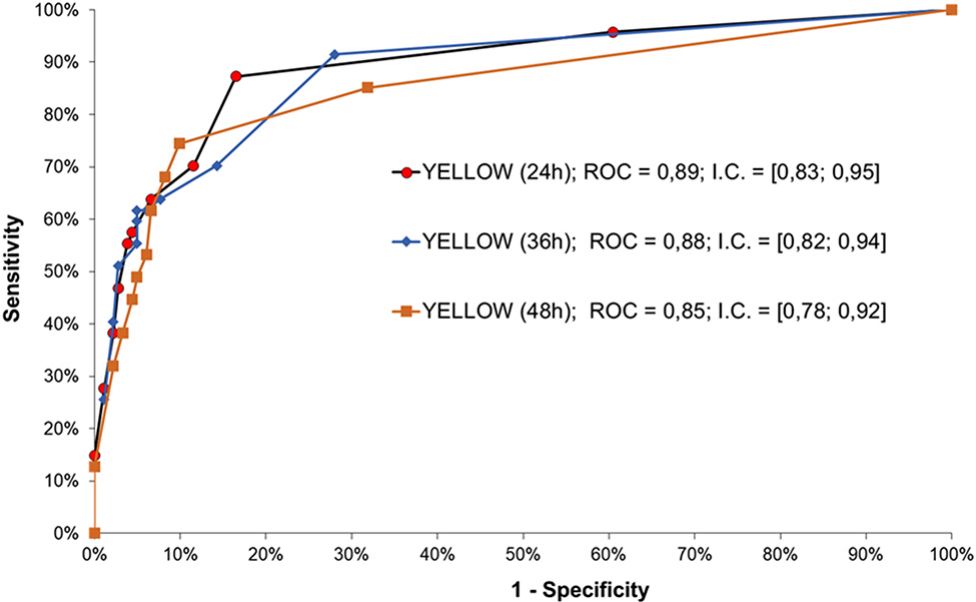
ROC (Receiver Operating Characteristic) curves evaluating the predictive performance of the three logistic regression Y.E.L.L.O.W. Score models. ROC (Receiver Operating Characteristic) curves evaluating the predictive performance of the three logistic regression Y.E.L.L.O.W. Score models.

**Results:**

Of 229 patients, 197 (86%) were male; median age was 47 (range: 18-82, SD: 13.4). Median time to hospitalization from symptom onset was 4 days (range: 0-40). Bleeding occurred in 41 patients (17.9%), and sepsis in 6 (2.6%). Transfusions were given to 64 (27.9%), and 26 (11.4%) needed dialysis. Multivariate analysis included: age, encephalopathy grade, hemoglobin, platelets, leukocytes, neutrophils, INR (International Normalized Ratio), AST (aspartate transaminase), direct bilirubin, creatinine, and lactate. Logistic regression identified 6 risk factors and 1 protective factor for YF death. To maintain consistency across time points (24, 36, and 48 hours post-admission), 7 variables were kept in models, even without significance (Table 1). Mortality probability is estimated from logistic regression models (QR code in Fig. 1). Fig. 2 shows spreadsheet examples for Y.E.L.L.O.W. Score mortality probability. All 3 models highly predict YF death (Fig. 3).

**Conclusion:**

This study developed and validated the Y.E.L.L.O.W. Score, a novel method for estimating death probability in yellow fever patients with acute liver failure. The score uses readily available clinical and laboratory data from 24, 36, and 48 hours post-admission. Logistic regression models show high predictive ability, suggesting the Y.E.L.L.O.W. Score is a valuable tool for clinicians in assessing prognosis and guiding management of yellow fever-related acute liver failure.

**Disclosures:**

All Authors: No reported disclosures

